# Fragment Merger: An Online Tool to Merge Overlapping Long Sequence Fragments

**DOI:** 10.3390/v5030824

**Published:** 2013-03-12

**Authors:** Trevor G. Bell, Anna Kramvis

**Affiliations:** Hepatitis Virus Diversity Research Programme (HVDRP), Department of Internal Medicine, School of Clinical Medicine, Faculty of Health Sciences, University of the Witwatersrand, 2050 Johannesburg, South Africa

**Keywords:** sequence data, sequence fragments, chromatograms, DNA assembly, ampli-cons, hepatitis B virus

## Abstract

While PCR amplicons extend to a few thousand bases, the length of sequences from direct Sanger sequencing is limited to 500–800 nucleotides. Therefore, several fragments may be required to cover an amplicon, a gene or an entire genome. These fragments are typically sequenced in an overlapping fashion and assembled by manually sliding and aligning the sequences visually. This is time-consuming, repetitive and error-prone, and further complicated by circular genomes. An online tool merging two to twelve long overlapping sequence fragments was developed. Either chromatograms or FASTA files are submitted to the tool, which trims poor quality ends of chromatograms according to user-specified parameters. Fragments are assembled into a single sequence by repeatedly calling the EMBOSS *merger* tool in a consecutive manner. Output includes the number of trimmed nucleotides, details of each merge, and an optional alignment to a reference sequence. The final merge sequence is displayed and can be downloaded in FASTA format. All output files can be downloaded as a ZIP archive. This tool allows for easy and automated assembly of overlapping sequences and is aimed at researchers without specialist computer skills. The tool is genome- and organism-agnostic and has been developed using hepatitis B virus sequence data.

## 1. Introduction

DNA sequencing is a routine procedure in many wet laboratories. This sequencing may include direct, Sanger sequencing, or any of the many “next generation” sequencing technologies. Many sequence assembly software programs are available [1,2,3,4,5,6]. Most of these programs assemble short reads of next-generation sequencing data, with a small number assembling a query sequence against a reference sequence (mapping assembly). Some of these programs are only available commercially, and some are only available on specific operating system platforms. Comprehensive, integrated bioinformatics software solutions, which may also include such functionality, are typically very expensive and their usage is restricted to licensed workstations only. Such software suites are often complex, requiring training and high levels of computer proficiency to operate effectively. In addition, installing and using many of the available programs can be difficult, may require technical expertise, access to a specific operating system platform or expensive hardware. In resource-limited settings, where capacity and finances are generally limited, the purchase and use of such software is not possible. To address some of these issues, we have developed an online, web-based, sequence assembly tool to easily assemble overlapping long PCR amplicons as sequenced by direct, Sanger sequencing technology. A web-based tool requires no installation and can be used via any web-browser from any operating system platform. No specialist technical skills are required to use the tool, which was developed and tested extensively using hepatitis B virus (HBV) sequence data.

HBV has a partially double-stranded circular DNA genome, which, depending on genotype, ranges in length from 3182 to 3248 nucleotides. By convention, the *EcoR*I restriction enzyme cleavage site (G*|*AATTC), located within the surface gene, is denoted as nucleotide position 1. The genome contains four partially overlapping reading frames and codes for seven proteins. Nine distinct HBV genotypes are known, with up to 32 subgenotypes described to date [7,8]. Sequence heterogeneity is common in HBV, as the viral polymerase lacks proof-reading ability [9,10]. Furthermore, variant strains, which exhibit insertions, deletions or SNPs, are commonly encountered and reported. In addition, recombination of large or small regions between two or more variants has also been reported [11].

Sequence data from either the surface (S) gene (approximately 1200 nucleotides) or the entire genome are essential to determine the viral genotype or subgenotype, to characterize the virus, and to identify indels and SNPs. Such data are routinely obtained by single or nested PCR amplification of the regions in question [12,13], followed by direct sequencing (typically in the forward direction only) using a number of internal sequencing primers. The resulting amplicons are typically between 500 and 800 nucleotides in length. The HBV genome is circular, but the resulting sequence is a linear fragment. Assembling these fragments into a complete gene or genome has previously been undertaken manually. The chromatogram (trace) file for each fragment is viewed [14,15] and checked. The poor quality ends are trimmed manually. Sequence data for each fragment is imported into an editor, such as GeneDoc [16], and each fragment is slid until it overlaps with another fragment. The complete sequence is then constructed by either entering the sequence of bases or by editing a reference sequence. This process is extremely time-consuming, repetitive and error-prone.

We describe here the implementation of a genome-agnostic, web-based, assembly tool, which has been developed using HBV sequence data.

## 2. Implementation

### 2.1. Overview

The online tool we have developed is a Python 2.6.5+ CGI script [17], hosted on an Ubuntu GNU/Linux server [18]. The tool processes between two and twelve overlapping long sequence fragments; that is, a series of fragments in which sequence data between subsequent pairs of fragments overlap. These fragments may be from either chromatograms (trace files in “AB1” format) or FASTA files, or a mixture of both formats. FASTA files (which will not be trimmed by the tool) would typically contain sequence data, which has previously been curated (trimmed and checked). Chromatograms will be trimmed according to the “trim window” and “trim threshold” integer parameters specified for each file. Base calls and quality scores are extracted from each chromatogram using a Python ABIF file reader [19]. A moving window of “trim window” nucleotides is slid over each sequence, from each end, until the quality scores of all nucleotides within this window are greater than or equal to the “trim threshold” value. The default values of 10 for the trim window and 20 for the trim threshold have proven to be suitable for the HBV sequence data analyzed during the development of the tool. After the chromatogram has been trimmed, the base calls in the chromatogram are extracted and used as the sequence data. If a chromatogram is of such poor quality that it is trimmed to a length of zero, the user will be notified and no merge will be performed. As the function of this tool is not to assemble shotgun sequence data, fragment files (chromatograms and/or FASTA files) must be specified in the order in which the fragments should be merged. Sequences, which should be reversed and/or complemented, must be specified before they are merged. This information is readily available from the amplification and sequencing protocols used.

The EMBOSS *merger* program [20], which implements the Needleman–Wunsch global alignment algorithm [21] to align two sequences, is used with default parameters to merge the sequence data. When only two input fragments are specified, the *merger* tool is executed from the Python script with two input files and the output is written directly to an output file. When more than two fragments are specified, a “bash” shell command is built, which calls the *merger* program repeatedly, depending on the number of fragments specified. One of the two input sequences to the *merger* tool can be provided from the UNIX “stdin” pipe and the output can be redirected to the UNIX “stdout” pipe. To avoid creating many temporary files for each of the successive two-fragment merges, the “bash” shell command is constructed such that the output from one merge is piped in as the input to the next merge. The following three commands are used to construct the required command-line:

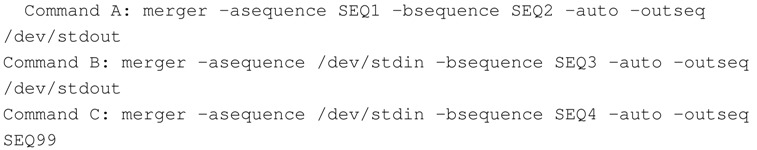


Command A runs the *merger* program to merge sequences *SEQ1* and *SEQ2* and writes the output to “stdout”. Command B merges the data from “stdin” with sequence *SEQ3* and writes the output to “stdout”. Command C merges the data from “stdin” with *SEQ4* and writes the output to an output file *SEQ99*, which will then contain the complete merged sequence. This process can therefore be generalised into a command-line consisting of “Command A — Command B (repeated *n −* 3 times) — Command C”, where “*n*” is the number of fragments and *n ≥* 3. For example, if three fragments are specified, the command-line will consist of “Command A — Command C”. Command B will not be included, as it is repeated (*n −* 3) times. If five fragments are specified, the command-line will consist of “Command A — Command B — Command B — Command C”. The total number of merges executed is therefore *n −* 1.

The *Fragment Merger Tool* consists of a web-based front-end (“client” interface) with which the user interacts, and a CGI (common gateway interface) script on a server, which runs Python language [17] code to generate the output web-page. The tool is one component of a larger project currently under development, which makes use of a common, shared Python library. The input files, which the user specifies, are saved locally (on the server) by the CGI script and then processed by the Python library. Methods within this library are responsible for loading sequence data from the files, processing the input parameters, and executing the merges. The output HTML page is written to disk by the Python script. The tool is an online resource, which requires a client browser to connect to the tool’s web-server, and therefore no stand-alone, offline version is available for download.

### 2.2. Input

**Figure 1:**
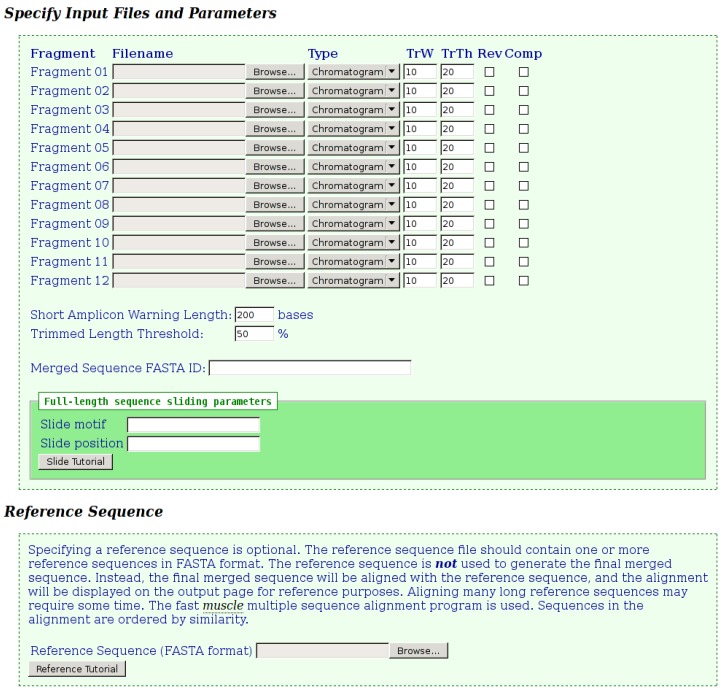
**Fragment Merger Input Page.** Each input file (chromatogram or FASTA file) is specified in the order in which the merge is to be performed. Trimming parameters can be specified for each chromatogram. Selecting the “Rev” or “Comp” check-boxes will reverse or complement the sequence data, respectively. Two input boxes specify the thresholds for considering a fragment as “short” and for triggering a warning for potentially poor quality chromatograms. Sliding parameters and a reference sequence file (in FASTA format) can be specified.

The input page for the tool is shown in Figure 1. Filenames for two to twelve chromatograms and/or FASTA files are specified. The “type” field will automatically change to indicate the type of the input file based on the filename extension, but can be changed manually, if required. When a chromatogram is specified, the trim window (“TrW”) and trim threshold (“TrTh”) values can be adjusted, as described previously. Relaxing these parameters may assist in obtaining a merged sequence, by less stringently trimming lower quality ends. Sequence data can be reversed and/or complemented as required, by selecting the appropriate check-boxes for each fragment. The tool will display a yellow “warning” or a red “error” icon on the output page next to fragments, which are below specified lengths, to assist the user in checking the input data. Fragments, which are shorter than the “Short Amplicon Warning Length”, will be flagged with the “warning” icon, while those that are trimmed to below the “Trimmed Length Threshold” percentage of their original length will be flagged with the “error” icon. The value entered into the optional “Merged Sequence FASTA ID” field will be used as the FASTA ID of the final merged sequence. If this field is omitted, the FASTA ID of the merged sequence will be that of the first fragment submitted. When sequences are trimmed, the trim parameters will be appended to the existing FASTA ID for reference.

Optional “Slide motif” and “Slide position” parameters may also be specified. These are only applicable when assembling full-length circular genomes. A final merged sequence representing a full genome is unlikely to be in the correct orientation because of the location of the various sequencing primers. However, the nucleotide data of such sequences can be slid into the correct orientation. For

HBV, for example, the *EcoR*I restriction enzyme cleavage site (G*|*AATTC), located within the surface gene, is denoted as nucleotide position 1. When a slide motif and slide position are specified, the tool will slide the final merged sequence so that the specified motif is located at the specified position in the sequence. If the quality or orientation of a merge is not satisfactory, the process can easily be repeated, after adjusting the relevant input parameters and/or specifying sliding parameters.

Optionally, a reference sequence file, containing one or more reference sequences in FASTA format, may be specified. These reference sequences are not used to construct the merged sequence, but to check the merged sequence using multiple sequence alignment as implemented by the fast Muscle program [22]. The alignment will be included on the output page and made available for download.

When fragments derived from a full-length circular genome (such as a viral genome) are specified, it is likely that the final merged sequence will not be in the correct orientation. The same scenario will occur when sequences straddling the “start” position of a circular genome are specified. In the former case, the merge sequence can be slid into the correct orientation by specifying appropriate “slide” parameters on the input page as described above. In either case, specifying a reference sequence in the standard orientation will result in an alignment with long regions of gaps on one or both ends. This can be avoided by preparing the reference sequence to match the orientation of the merged sequence, or by preparing a double-length reference sequence. Such a sequence would place the end of the first linear sequence adjacent to the start of the second linear sequence, thereby creating an artificial full-length linear sequence.

## 3. Results and Discussion

**Figure 2:**
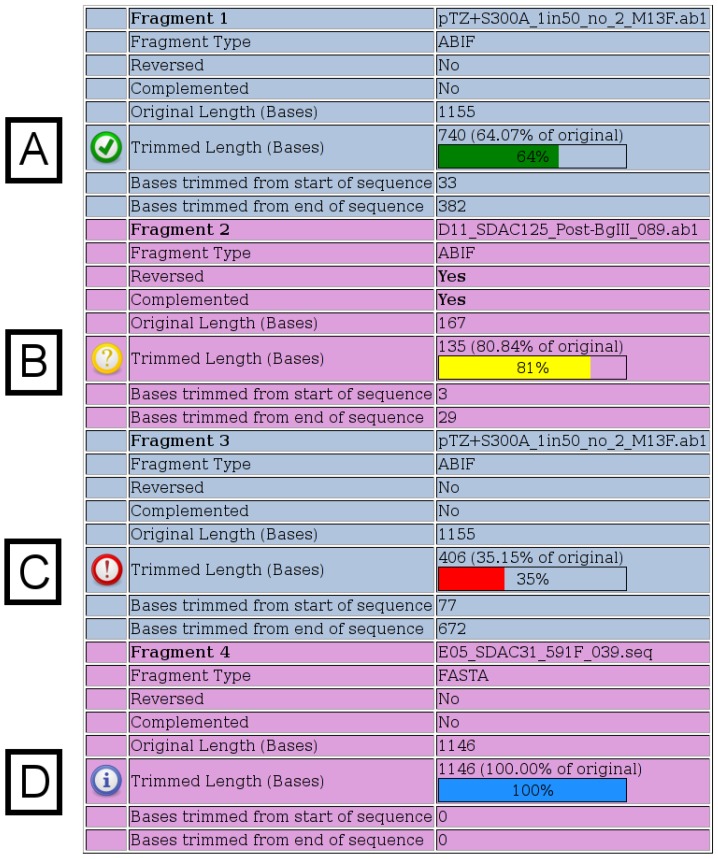
**Fragment Details.** Details for each fragment submitted are shown on the output page. All possible notification icons are shown here. (**A**) A green icon indicates that the chromatogram has been trimmed, but is not shorter than any of the specified thresholds. (**B**) A yellow icon indicates that the trimmed chromatogram is shorter than the specified warning length, which has a default value of 200. (**C**) A red icon indicates that the trimmed chromatogram is shorter than the specified percentage of its original length, which has a default value of 50%. (**D**) A blue icon indicates that a FASTA file was specified, in which case, no trimming is performed.

**Figure 3:**
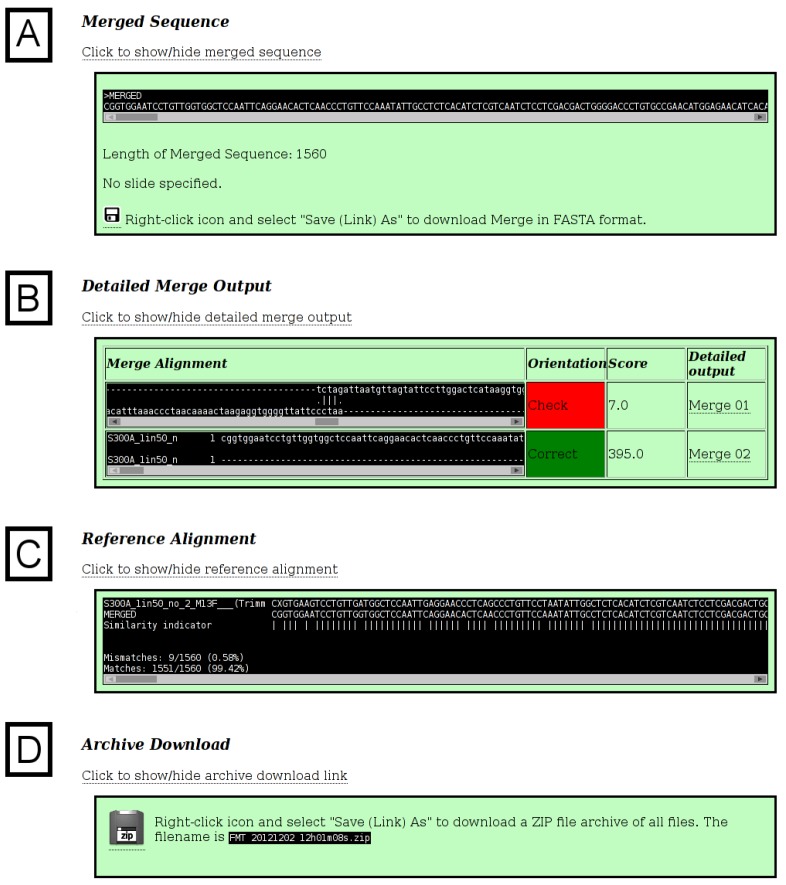
**Additional Output.** The additional sections of the output page are shown. (**A**) The final merged sequence in FASTA format is displayed and can be downloaded. (**B**) The detailed output of each of the successive merges is provided in a table. The alignment of the two fragments for each merge, and their score (as generated by the *merger* program), are provided. Since the fragments are merged in order sequentially, the expectation is that the end of the first fragment in each merge will overlap with the start of the next fragment. If this is the case, a green table cell with the word “Correct” is shown under the “Orientation” column. If the merge has occurred in the other orientation, the cell is shaded red with the word “Check” displayed. The final column provides a hyperlink to the full, detailed output for each merge, as generated by the *merger* program. This output includes a table detailing any conflicts, which the *merger* program detected, between the two sequences, and the base, which was used in the output sequence. It is advisable to check this detailed output before continuing to use the final merged sequence in any downstream applications or analyses. (**C**) The alignment of the merged sequence against the reference sequence(s) is shown. Conserved loci across all sequences are indicated with a “*|*” character, mismatches are indicated with a space character, and the total number of matches and mismatches is shown. (**D**) An archive containing all input and output files (excluding chromatograms) can be downloaded. All the files in the archive are in a folder named with the date and time the merge was executed.

**Figure 4:**
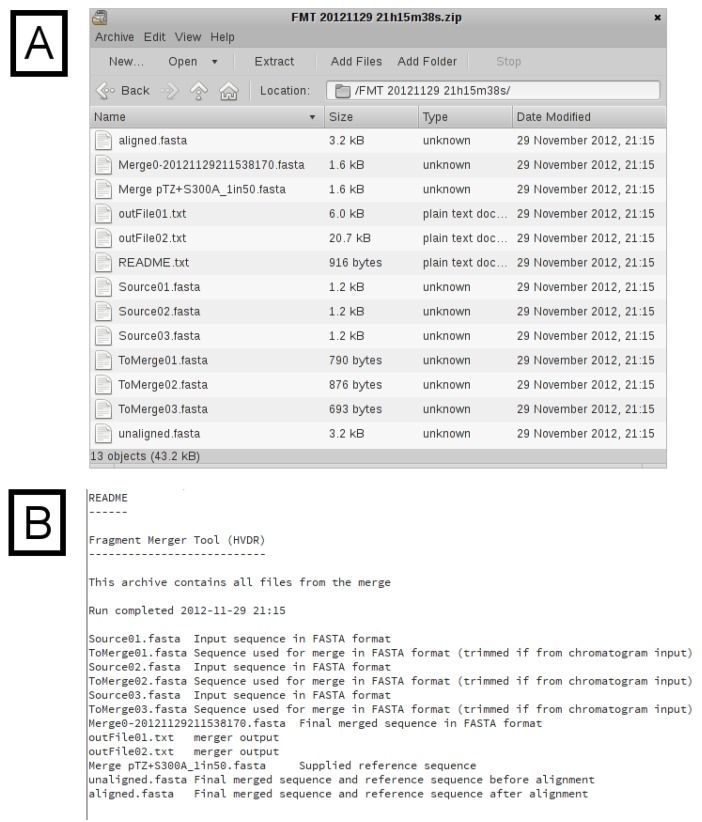
**Archive Contents.** (**A**) A list of the files in the archive. (**B**) The “README.txt” file from the downloaded archive. This file provides the filename and a description for all of the files in the archive.

Once the merges have run, detailed output is provided to the user as shown in Figure 2, Figure 3, Figure 4. The first section of output (Figure 2) displays details for each fragment submitted. When chromatograms are submitted, the number of bases, which were trimmed from each end, is shown, whereas when FASTA files are submitted, no trimming is performed and a value of “100%” is shown. The various possible notification icons are described in Figure 2. Data from fragments flagged with a yellow or red icon are not excluded from the merge, but the user should check the final merged sequence carefully.

The additional sections of output are shown in Figure 3, Figure 4. In Figure 3A, the final merged (assembled) sequence is shown and can be downloaded as a file in FASTA format. The length is displayed, as well as any sliding parameters, which were provided. Detailed output of each of the successive merges, as generated by the *merger* program, is provided in a table (Figure 3B). If a reference sequence file was specified, the merged sequence aligned against the reference sequence/s is displayed (Figure 3C). The alignment is intended to be used as a quick check to validate the success and/or accuracy of the merge. If no merge is possible (because of insufficient areas of overlap between two sequences), the *merger* program will simply concatenate the two input sequences, with only one overlapping nucleotide. It is therefore important that the final sequence is checked carefully, preferably against known reference sequences. If the reference sequence file contains several sequences, additional time will be required to generate the alignment. Typically, one or two reference sequences should be sufficient. A download hyperlink to a ZIP archive file containing all input and output files (excluding chromatograms) is provided. The archive (Figure 4A) contains the untrimmed input sequence data, the trimmed input sequence data (files starting with the name “ToMerge”), the final merged sequence (a file starting with the name “Merge0”), the reference sequence file, the unaligned merged and reference sequences, the aligned merged and reference sequences, the output text files from the *merger* program for each merge (files starting with the name “outFile”) and a “README” text file describing each of the files, for reference (Figure 4B).

The time taken for the Python CGI script to execute was calculated by subtracting the timestamp when the script completed from the timestamp when the script started. The average execution time, from 195 runs of the tool over several months, merging three overlapping fragments from chromatogram input data, was 0.48*±*0.12 seconds.

The tool has been used extensively to assemble the complete surface gene of HBV from three overlapping fragments. Although the HBV genome is circular, sequence data (either from direct

sequencing or from sequencing of clones) is linear. The tool can therefore be used with both circular and linear sequences. The tool has also been used to assemble entire HBV genomes from 6 or 7 overlapping fragments. In this case, fragments in both the forward and reverse direction were used.

The tool has been designed to assist users in processing sequence data and requires that the user exercise discretion when submitting data and interpreting the results. Poor quality data in chromatograms

could result in false indels in the final sequence. The tool does not disambiguate any ambiguous bases in chromatogram data, nor does it search for, or remove, any vector-specific or other primer sequences. If this is required, a FASTA file of the edited data, without the primer sequences, should be submitted to the tool. This is a not a mapping assembler and does not make use of a reference sequence for assembly.

## 4. Conclusions

This tool allows for easy and automated assembly of long overlapping sequence fragments, which can be input as either chromatograms or FASTA files, and provides detailed visual output. Sequence data from insertion or deletion mutants and recombinants can be assembled, as a reference sequence is not used for assembly. Although it has been developed and tested extensively on HBV sequence data, it is genome- and organism-agnostic. The length of the final, merged sequence should be less than 100,000 nucleotides. The tool does not require technical expertise, or special hardware or software to use, and is aimed at researchers without specialist computer skills.
